# Fungal Infections in Kidney Transplant Recipients: A Comprehensive Narrative Review

**DOI:** 10.3390/microorganisms13010207

**Published:** 2025-01-18

**Authors:** Maria Mazzitelli, Federico Nalesso, Alberto Enrico Maraolo, Vincenzo Scaglione, Lucrezia Furian, Annamaria Cattelan

**Affiliations:** 1 Infectious and Tropical Diseases Unit, Padua University Hospital, 35128 Padua, Italy; vincenzo.scaglione@aopd.veneto.it (V.S.); annamaria.cattelan@unipd.it (A.C.); 2 Nephrology, Dialysis and Transplantation Unit, Department of Medicine, University of Padova, 35128 Padova, Italy; federico.nalesso@unidp.it; 3 Section of Infectious Diseases, Department of Clinical Medicine and Surgery, University of Naples “Federico II”, 80131 Naples, Italy; albertomaraolo@mail.com; 4 Kidney and Pancreas Transplantation Unit, Padua University Hospital, 35128 Padua, Italy; lucrezia.furian@unipd.it; 5 Department of Molecular Medicine, University of Padova, 35128 Padua, Italy

**Keywords:** fungal infection, *Candida*, *Aspergillus*, *Pneumocystis*, kidney transplant, kidney transplant recipients, complications

## Abstract

Background: Despite kidney transplantation being a life-saving procedure, patients experience a high risk of developing fungal infections (FIs), with an increased risk of both morbidity and mortality, especially during the first year after transplant. Methods: We herein conducted a narrative review of the most common FIs in kidney transplant recipients (KTRs), with a focus on prevalence, risk factors, mortality, and prevention strategies. Results: The most common fungal pathogens in KTRs include *Candida* species (up to 70% of the overall FIs), *Aspergillus* species, *Pneumocystis jiroveci*, and *Cryptococcus* species. Fungal colonization, diabetes mellitus, chronic liver disease, malnutrition, and pre-existing lung conditions should all be acknowledged as possible predisposing risk factors. The mortality rate can vary from 25 to 50% and according to different settings and the types of FIs. Preventive strategies are critical for reducing the incidence of FIs in this population. These include antifungal prophylaxis, environmental precautions, and infection control measures. The use of novel tools (such as PCR-based molecular assays and NGS) for rapid and accurate diagnosis may play an important role. Conclusions: Early recognition, the appropriate use of antifungal therapy, and preventive strategies are essential for improving graft loss and fatal outcomes in this vulnerable population. Future research is needed to optimize diagnostic tools, identify novel antifungal agents, and develop better prophylactic strategies for high-risk transplant recipients.

## 1. Background

Kidney transplantation is a life-saving procedure for patients with end-stage renal disease (ESRD), offering improved quality of life and longevity [1,2]. However, the whole process, including the immunosuppressive therapy necessary to prevent graft rejection, significantly increases the risk of opportunistic infections, including fungal infections (FIs) [3,4]. FIs represent a significant cause of morbidity and mortality in kidney transplant recipients (KTRs) [5]. These infections arise due to the profound immunosuppressive therapy required to prevent organ rejection, which compromises the immune system and leaves the patient susceptible to opportunistic fungal pathogens [6]. Fungal infections in KTRs are less common than bacterial or viral infections, yet they carry a high risk of morbidity, mortality, and graft loss [7,8]. Opportunistic fungal infections can range from superficial to life-threatening systemic infections [9]. The aim of this narrative review is to provide a comprehensive overview of FIs in KTRs, focusing on epidemiology, pathogenesis, risk factors, common fungal pathogens, clinical presentation, diagnostic approaches, treatment strategies, and preventive measures.

## 2. Epidemiology of Fungal Infections in Kidney Transplant Recipients

FIs in KTRs have a lower incidence compared to bacterial and viral infections, but they are responsible for a disproportionately high rate of complications and mortality [9,10]. The incidence of fungal infections in kidney transplant recipients is estimated to range from 5% to 20%, depending on the geographical region, type of immunosuppression, and post-transplant care protocols [11]. They account for approximately 5–10% of infectious complications after kidney transplantation, with an associated mortality rate of 25–50% in cases of invasive fungal infections (IFIs) [12,13,14]. The incidence of fungal infections varies geographically, with certain endemic fungi being more prevalent in specific regions. For instance, *Histoplasma capsulatum* is more commonly found in the Ohio and Mississippi River valleys in the United States, while *Coccidioides immitis* is endemic to the southwestern U.S. and parts of Latin America [15,16]. In contrast, *Aspergillus* and *Candida* infections are seen globally, with *Candida* species being the most common cause of fungal infections in KTRs, accounting for 50–70% of cases [13]. FIs in KTRs can occur in three different temporal post-transplant periods: early (within 1–3 months), intermediate (3–12 months), and late (beyond 12 months) [14]. The incidence of fungal infections tends to be highest in the first six months post-transplant when immunosuppressive therapy is most intense, although late-onset fungal infections can also occur [14,17]. Early fungal infections are often nosocomial, linked to surgical procedures, the use of central venous catheters, or prolonged antibiotic use [11,13]. Intermediate infections are often related to the cumulative effects of immunosuppression, while late fungal infections are frequently due to environmental exposure or the reactivation of latent infections, such as cryptococcosis [11,13]. Further details about each specific fungal species are reported below.

## 3. Pathogenesis and Risk Factors for Fungal Infections

The pathogenesis of FIs in KTRs is influenced by several factors, such as the type of fungal pathogen, the host’s immune status, and environmental factors [18]. Opportunistic fungal pathogens, which are typically low-virulence organisms, become pathogenic in immunocompromised individuals [19]. Overall, risk factors for FIs may be broadly categorized into immunological, procedural, and environmental factors. Among immunological factors, we can list immunosuppressive therapy, cumulative immunosuppression, and prolonged neutropenia [20]. The cornerstone of post-transplant management is immunosuppression to prevent graft rejection. However, this therapy severely impairs the immune response, increasing the risk of opportunistic infections [21]. Calcineurin inhibitors (e.g., tacrolimus and cyclosporine), corticosteroids, and anti-proliferative agents such as mycophenolate mofetil or azathioprine all suppress immune function, particularly T-cell-mediated immunity, making patients vulnerable to FIs [21,22]. Moreover, the intensity and duration of immunosuppressive therapy directly correlate with the risk of infection [23]. Patients who require high doses of immunosuppressants due to acute rejection episodes are at heightened risk [24]. Although less common in KTRs compared to hematopoietic stem cell transplant recipients, episodes of neutropenia, particularly during treatment for rejection, can predispose patients to FIs, particularly invasive aspergillosis. Among procedural factors, we can list surgical complications, dialysis dependence, and prolonged antibiotic use [25]. Postoperative complications, including wound infections, urinary leaks, and the use of invasive devices like urinary catheters and central venous lines, can provide a portal of entry for fungal pathogens [25]. Patients who require prolonged dialysis before transplantation or who have delayed graft function post-transplant are at increased risk for infection, as dialysis can lead to bacterial and fungal colonization, which may subsequently develop into IFIs. The prolonged use of broad-spectrum antibiotics, which is common in the perioperative period, can disrupt the normal flora, allowing for fungal overgrowth, particularly of *Candida* species. However, despite this process being well known, to date, no guidelines have recommended the use of probiotics in the prevention or management of Fis.

**Figure 1:**
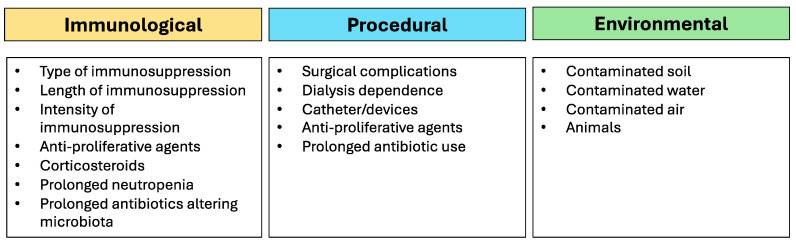
Summary of risk factors for fungal infection in kidney transplant recipients.

Previous studies demonstrated how different combinations of immunosuppressant agents in transplant recipients significantly changed the quantities of bacteria including *Ruminococcaceae*, *Lachnospiraceae*, *Firmicutes*, *Bacteroides*, and *Clostridiales*, at the same time increasing the risk of being colonized by *Escherichia coli* and *Enterococcus* spp. [26]. Also, changes in the urinary microbiome, induced by antibiotics and immunosuppression, may lead to chronic allograft dysfunction and poorer outcomes [27]. Pre-existing colonization may play an important role in the development of FIs [28]. Indeed, patients may be colonized with fungal organisms such as *Candida* in the gastrointestinal or respiratory tracts prior to transplantation [28]. This colonization can serve as a source of subsequent invasive infection. KTRs living in areas endemic for certain fungi, such as *Histoplasma* or *Coccidioides*, are at increased risk for primary infection or the reactivation of latent infections [29,30]. Among other predisposing factors, diabetes mellitus, chronic liver disease, malnutrition, and pre-existing lung conditions (e.g., bronchiectasis or chronic obstructive pulmonary disease) should all be acknowledged as possible predisposing risk factors for fungal infections in KTRs [3,6]. Regarding environmental factors, the places where patients live play a fundamental role in risk assessment. Indeed, airborne fungi or their products may come from different natural sources such as soil, lakes, plants, and animals, as well as from human activities such as sewage treatment, fermentative processes, animal rendering, and agricultural activities [31,32]. Risk factors for fungal infection are depicted in Figure 1.

## 4. Major Fungal Pathogens in Kidney Transplant Recipients

The most common fungal pathogens in KTRs include *Candida* species, *Aspergillus* species, *Pneumocystis jiroveci*, and *Cryptococcus* species [10,17,33]. In addition, endemic fungi such as *Histoplasma capsulatum*, *Coccidioides immitis*, and *Blastomyces dermatitidis* can cause infections in specific geographical regions [15,16,29,30,34]. The main features of each FI are summarized in Table 1.

### 4.1. Candida spp.

Candidiasis is the most common FI in KTRs, accounting for up to 70% of FIs in this population [10]. The infection can present as superficial mucocutaneous disease (oral or anogenital/vaginal candidiasis) or as a more serious invasive disease involving the bloodstream (candidemia), kidneys (fungal pyelonephritis), or intra-abdominal organs [9,33]. Invasive *Candida* infections, particularly candidemia, are associated with significant morbidity and mortality [33]. Non-*albicans Candida* species, such as *Candida glabrata* and *Candida krusei*, are increasingly reported in KTRs and are often resistant to standard antifungal therapy [35]. It is interesting to notice that in the case of KTRs, there is a significant difference in the frequency of infections caused by *Candida albicans* and non-albicans species. Risk factors for *Candida* infection include the prolonged use of broad-spectrum antibiotics, high-dose corticosteroids, the use of central venous catheters, and prior colonization with *Candida* species [33]. Invasive *Candida* infections often manifest as fever, hemodynamic instability, and evidence of multi-organ dysfunction, but quite often can be too insidious to be recognized [9,33]. Candidemia is the most common form of invasive disease and can lead to complications such as endocarditis, osteomyelitis, and septic emboli [36]. Candiduria is a frequent finding after kidney transplantation (KT) but is rarely clinically significant, typically occurring around a median of 25 days post-transplant, often affecting diabetic female patients with substantial prior exposure to antimicrobials [37]. Studies have demonstrated that antifungal treatment for asymptomatic candiduria does not reduce the risk of recurrence, facilitate urinary clearance, or prevent severe complications [38]. On the other hand, a true urinary tract infection such as fungal pyelonephritis can present with fever, flank pain, and graft dysfunction in KTRs. Diagnosis is established by blood cultures, tissue biopsies, or urine cultures in the case of pyelonephritis [36]. *Candida albicans* is the most frequently isolated species, but non-*albicans* species such as *Candida glabrata* and *Candida krusei*, which are more resistant to standard antifungal agents, are increasingly reported [35,36]. The detection of a *Candida* antigen and the use of molecular diagnostic tools such as polymerase chain reaction (PCR) have improved the speed and accuracy of diagnosis [33]. First-line treatment for invasive *Candida* infections includes echinocandins (e.g., caspofungin, micafungin, anidulafungin) or liposomal amphotericin B [33]. Fluconazole is effective for susceptible strains of *Candida albicans*, but resistance is a growing concern, especially in non-*albicans* species [39].

### 4.2. Aspergillus spp.

Aspergillosis is the second-most common and severe cause of FIs in KTRs, with a high mortality rate, especially in the setting of pulmonary involvement [10]. Most infections are caused by *Aspergillus fumigatus*, although other species such as *Aspergillus flavus* and *Aspergillus terreus* have also been implicated and described [40,41]. Compared to in other solid organ transplant patients, the incidence of Aspergillosis in KTRs is lower, with a described incidence of 0.4% [42]. Risk factors for invasive aspergillosis include high-dose immunosuppression, neutropenia, chronic lung disease, and exposure to construction sites or other environments contaminated with fungal spores. Pulmonary aspergillosis is the most common manifestation and typically presents with fever, cough, dyspnea, and hemoptysis [42]. In some cases, it can cause cavitary lung lesions, which may be mistaken for bacterial pneumonia [43]. Disseminated aspergillosis can affect the brain, liver, and kidneys, often leading to severe complications [44]. The diagnosis of invasive aspergillosis requires a high index of suspicion and can be confirmed through cultures, histopathology, or the detection of the galactomannan (GM) antigen in serum or bronchoalveolar lavage fluid [45,46].

Computed tomography (CT) of the chest often shows nodules or cavitary lesions [47]. First-line treatment includes voriconazole or isavuconazole [45,48]. Liposomal amphotericin B is an alternative option for patients who cannot tolerate azoles or in cases of resistant *Aspergillus* strains [48]. In refractory cases or cases of azole-resistant *Aspergillus*, combination therapy with echinocandins may be considered [48]. The duration of antifungal therapy depends on the severity of the infection and the patient’s clinical response [48]. For IFIs, prolonged therapy (6–12 weeks) is often required, followed by maintenance therapy with oral azoles to prevent relapse [49].

### 4.3. Pneumocystis jirovecii

*Pneumocystis* pneumonia (PCP) is a serious opportunistic infection caused by *Pneumocystis jiroveci* (*P. jirovecii*), a pathogen predominantly affecting immunocompromised individuals [50]. Among KTRs, the risk of PCP is significantly elevated due to the immunosuppressive medications required to prevent organ rejection [51]. These drugs, including calcineurin inhibitors, corticosteroids, antimetabolites, and newer immunosuppressive agents such as belatacept, suppress the immune system, making patients more susceptible to infections like *P. jirovecii*. Historically, PCP was a major cause of morbidity and mortality in transplant recipients, particularly those undergoing kidney transplants [52]. Although advances in prophylaxis and treatment have reduced its incidence, the infection still poses a serious risk, particularly in the first six to twelve months post-transplant when immunosuppression is most intense [53]. In a big retrospective cohort, its prevalence was 2.1%, with 54% and 14% developing a very severe form and dying, respectively [54]. Other risk factors for PCP include the presence of graft rejection, the use of high-dose steroids, and concurrent infections [55]. In KTRs, PCP often presents with non-specific respiratory symptoms, including cough, fever, and shortness of breath. These symptoms may progress rapidly, leading to respiratory failure if not promptly diagnosed and treated. In contrast to immunocompetent individuals, presentation in transplant recipients can be more insidious, with subtle symptoms that may be mistaken for other common post-transplant complications, such as bacterial or viral infections [56]. Additionally, *P. jirovecii* can cause extra-pulmonary manifestations, although these are less common [57].

Diagnosing PCP in KTRs can be challenging due to its non-specific clinical features. Diagnostic methods typically include the detection of *P. jirovecii* DNA in respiratory samples using polymerase chain reaction (PCR) testing or microscopic identification in bronchoalveolar lavage (BAL) fluid [56]. Imaging, such as chest X-rays or CT scans, often reveals diffuse bilateral infiltrates [58].

To prevent PCP, prophylaxis with trimethoprim–sulfamethoxazole (TMP-SMX) is the standard of care for KTRs, particularly during the first six months post-transplant. Prophylaxis may extend beyond this period in patients with ongoing risk factors, such as those receiving high-dose corticosteroids [48]. Alternative prophylactic agents, such as dapsone or atovaquone, may be used in cases of TMP-SMX intolerance [48].

Once diagnosed, treatment involves high-dose TMP-SMX alongside adjunctive corticosteroids for severe cases to reduce the inflammation caused by the infection [48]. Despite effective treatment, mortality rates for PCP in KTRs can be high, particularly when diagnosis is delayed. Early recognition, prompt treatment, and prophylaxis are therefore crucial for reducing risk and improving outcomes in this vulnerable population [48].

### 4.4. Cryptococcus spp.

Cryptococcosis is caused by *Cryptococcus neoformans* and *Cryptococcus gattii*, encapsulated yeasts found in the environment, particularly in soil contaminated with bird droppings [59]. The infection most commonly affects the central nervous system (CNS) and lungs but can also involve other organs in disseminated disease [60]. The use of calcineurin inhibitors and the use of high-dose corticosteroids are significant risk factors for cryptococcosis in KTRs [61]. In solid organ transplant recipients, its prevalence ranges from 0.2% to 5%, but for KTRs, prevalence was described as being around 0.32% [62]. More recent data have shown a lower incidence of 0.04% [62]. Late-onset cryptococcal infections (>12 months post-transplant) are more common and may be related to both the reactivation of latent infection and the discontinuation of prophylaxis [63]. Latent infection has been shown to be present in up to 52% of transplant recipients who then develop cryptococcosis [64]. Cryptococcal meningitis is the most common presentation in transplant recipients, with symptoms including headache, fever, altered mental status, and photophobia [60]. In a large cohort study from Taiwan matching 4933 KTRs and 49,930 non-KTR subjects, the cryptococcosis incidence rates for the former group and the latter group were 10.59 and 0.4 per 10,000 person-years, respectively, with the hazard ratio (HR) for central nervous system involvement in the KTRs being 43.8 (*p* < 0.001) [65]. In this cohort, advanced age and concurrent cancer were found to be strong predictors of cryptococcosis among KTRs [X3]. Pulmonary cryptococcosis may mimic bacterial or viral pneumonia, with cough, chest pain, and dyspnea. Mortality ranges from 33 to 40% and worsens with more severe central nervous system involvement [62]. Diagnosis is made by detecting the cryptococcal antigen in the cerebrospinal fluid (CSF) or serum, as well as through culture or histopathological examinations. Imaging of the brain may show hydrocephalus or other CNS abnormalities. First-line treatment involves liposomal amphotericin B combined with flucytosine (which is not extensively available in different clinical settings), followed by fluconazole for long-term maintenance therapy [66].

### 4.5. Endemic Fungi

Endemic fungal infections, such as histoplasmosis, coccidioidomycosis, and blastomycosis, are rare but important causes of infection in KTRs, particularly in patients residing in or traveling to endemic areas. Data about mortality in the specific setting of KTRs are poor, but rates may reach 50% [67].

*Histoplasma capsulatum* is prevalent in the Ohio and Mississippi River valleys, Central and South America, and areas of Africa and Asia, where it is found in soil contaminated with bird or bat droppings [68]. Its prevalence in the setting of solid organ transplants is very heterogeneous due to a lack of access to available diagnostics and infection recognition, as well as insufficient surveillance and reporting [69]). However, a 0.1% 1-year cumulative incidence of histoplasmosis in all SOTs was reported [70]. Infection occurs via the inhalation of spores, leading to pulmonary disease, which may disseminate in immunocompromised patients [67]. Histoplasmosis can present as a subacute respiratory illness with fever, cough, and chest pain. Disseminated disease may involve the liver, spleen, bone marrow, and gastrointestinal tract [67]. Diagnosis is based on fungal culture, serology, and antigen detection in urine or serum [71]. When it occurs within the first months, especially in the first month, after transplant, a donor-derived infection should be suspected [72,73].

Treatment typically involves liposomal amphotericin B followed by itraconazole for maintenance therapy [71].

*Coccidioides immitis* is endemic to the southwestern U.S. and parts of Latin America [3,10,16]. The incidence of Coccidioidomycosis in KTRs in endemic areas may reach up to 3% [74]. Infection occurs via the inhalation of arthroconidia from soil [16,67]. Coccidioidomycosis presents with flu-like symptoms, including fever, cough, and fatigue [67,74]. Disseminated disease can involve the skin, bones, and central nervous system. Serologic tests, fungal culture, and PCR are used for diagnosis [67,74]. Treatment involves fluconazole or itraconazole for mild cases and liposomal amphotericin B for severe disease [75].

*Blastomyces dermatitidis* is endemic to the Mississippi and Ohio River valleys and the Great Lakes region [76]. The incidence of blastomycosis in KTRs in endemic areas is about 1% [77]. Blastomycosis typically presents as a respiratory illness with fever, cough, and weight loss [70]. Skin lesions and osteomyelitis are common in disseminated disease [78]. Diagnosis is based on fungal culture or histopathological examination of tissue biopsies [76]. Treatment involves liposomal amphotericin B for severe cases and itraconazole for mild to moderate disease [76].

## 5. Diagnostic Approaches

The early diagnosis of FIs in KTRs is challenging due to the non-specific nature of symptoms and overlapping clinical presentations with bacterial or viral infections [79]. A high index of suspicion is required, especially in patients with risk factors for FIs. Imaging studies using techniques such as CT and MRI are often useful in identifying fungal lesions, particularly in cases of pulmonary or central nervous system involvement [79].

Blood cultures are the gold standard especially for *Candida* spp. fungemia, and fungal culture remains, in general, the gold standard for the diagnosis of most FIs, although they may be time-consuming and may not always yield positive results [79,80,81]. Histopathological examination of tissue biopsies can provide a rapid diagnosis in some cases [82].

The limited sensitivity of blood cultures for diagnosing invasive candidiasis, particularly in deep-seated infections without candidemia, has prompted the development of alternative diagnostic methods targeting components of the fungal cell wall. Tests for 1-3-β-D-glucan, Candida mannan, and circulating IgG anti-Candida mannan antibodies have been evaluated over the past few decades, yielding variable outcomes in solid organ transplant recipients [33]. Unfortunately, specific large experiments in KTRs are lacking [33].

The detection of fungal antigens, such as GM for *Aspergillus* and the cryptococcal antigen for *Cryptococcus*, is useful for early diagnosis and/or for monitoring the response to treatment [81,83]. Indeed, despite its variable sensitivity observed across several studies for diagnostic purposes, GM could serve as a prognostic indicator in KTRs with invasive aspergillosis: for example, Heylen (2015) demonstrated that a GM index with an optical density >2 correlates with increased patient mortality [84]. This finding is reinforced by a retrospective study conducted at a tertiary-care referral hospital in Korea, which analyzed cases of invasive pulmonary aspergillosis (IPA) in renal transplant recipients from 1995 to 2015 [85]. The study found that a serum GM index >2 and a bronchoalveolar lavage (BAL) GM index >5.0 were associated with significantly higher 12-week mortality rates [85].

PCR-based assays and next-generation sequencing (NGS) are emerging tools for the rapid and accurate diagnosis of fungal infections, particularly for detecting rare or resistant fungal pathogens [81,83]. As for Candida spp., another possibility is represented by T2 Magnetic Resonance (T2MR) technology, which combines nuclear magnetic resonance with a PCR-based molecular assay to directly detect and identify Candida species from whole blood. The T2MR system can identify *Candida albicans*, *Candida glabrata*, *Candida parapsilosis*, *Candida tropicalis*, and *Candida krusei*, showing good sensitivity (around 90%) and excellent specificity (99%), with the restricted panel of pathogens detected being the most important limitation [33].

With regard to pneumocystosis, diagnostic data in KTRs are scarce, but it is worthwhile to mention a case series of six patients in which 1-3-β-D-glucan had 100% sensitivity in the early diagnosis of the FI [86].

The peculiarities of cryptococcosis diagnosis in KTRs have not been extensively studied. A small case series of three patients does not highlight particular differences compared with the diagnostic process for other types of patients [87].

## 6. Treatment Strategies, Antifungal Agents, Drug–Drug Interactions, and Toxicity

The treatment of FIs in KTRs is complicated by drug–drug interactions (DDIs) with immunosuppressive agents, nephrotoxicity, and the potential for drug resistance [88]. The choice of antifungal therapy depends on the specific pathogen, the site of infection, and the severity of disease [33,45,66,73,75,76]. Combination antifungal therapy may be required in severe or refractory cases. Liposomal amphotericin B is the most widely used polyene and is effective against a broad range of fungi, including *Candida*, *Aspergillus*, and endemic fungi [89,90]. It is often used as first-line therapy for severe or disseminated fungal infections, having the advantage of very limited DDIs. However, it is associated with significant nephrotoxicity, which can be problematic in KTRs [90]. Triazoles such as fluconazole, voriconazole, itraconazole, and posaconazole are commonly used for the treatment and prophylaxis of fungal infections in KTRs. Voriconazole is the drug of choice for invasive aspergillosis, while fluconazole is used for susceptible *Candida* infections and cryptococcosis [91]. Most recently, isavuconazole has been shown to be a good option in terms of efficacy, tolerability, and safety [91,92]. Caspofungin, micafungin, and anidulafungin are effective against *Candida* and *Aspergillus* species, although they have limited activity against *Cryptococcus* and endemic fungi [93]. Echinocandins are well tolerated and have a lower risk of nephrotoxicity compared to polyenes and azoles [93]. Antifungal drugs, particularly azoles, are potent inhibitors of the cytochrome P450 (CYP) enzyme system, leading to significant interactions with calcineurin inhibitors (e.g., tacrolimus and cyclosporine) and mTOR inhibitors (e.g., sirolimus and everolimus) [94]. The close monitoring of drug levels is essential to avoid toxicity or subtherapeutic immunosuppression in some cases, such as with voriconazole [91].

## 7. Prevention of Fungal Infections and Antifungal Prophylaxis

Preventive strategies are critical for reducing the incidence of fungal infections in KTRs. These include antifungal prophylaxis, environmental precautions, and infection control measures [95].

Antifungal prophylaxis may be considered for high-risk KTRs, particularly those with risk factors such as prolonged immunosuppression, neutropenia, or exposure to endemic fungi. Prophylactic agents include fluconazole, voriconazole, and echinocandins, depending on the specific risk factors and the local epidemiology of Fis [8]. However, it is noteworthy to mention that KTRs have a lower risk of developing Fis compared to other solid organ transplant recipients, such as those receiving livers, hearts, and lungs [85]. Indeed, broad-spectrum mold-active prophylaxis is not recommended; instead, PCP prophylaxis is recommended, especially in the early phase post-transplant [85]. KTRs should be advised to avoid activities that may expose them to fungal spores, such as gardening, handling soil, or visiting construction sites. In endemic areas, patients should avoid exposure to activities that may release fungal spores into the air, such as cave exploration or cleaning bird or bat droppings. Strict adherence to infection control practices in healthcare settings, including the use of sterile techniques during surgery and catheter insertion, is essential for preventing nosocomial FIs.

## 8. Conclusions

FIs in KTRs are a significant cause of morbidity and mortality, especially during the early phase in the post-transplant period [42]. The incidence of FIs and the number of cases observed are expected to increase in the coming years, also due to the annual increase in the number of transplants. Although less common than bacterial or viral infections, fungal infections often present as severe, invasive disease and can lead to graft loss or death if not promptly diagnosed and treated [8]. Early recognition, the appropriate use of antifungal therapy, and preventive strategies are essential for improving outcomes in this vulnerable population. Future research is needed to optimize diagnostic tools, identify novel antifungal agents, and develop better prophylactic strategies for high-risk transplant recipients.

## Figures and Tables

**Table 1:** Features of main fungal infections in kidney transplant recipients, from risk factors to treatment.

Pathogen	Risk Factors	Prevalence/Incidence	Mortality Rate	Diagnosis	Treatment
*Candida* spp.	Prolonged use of broad-spectrum antibiotics, high-dose corticosteroids, use of central venous catheters, and prior colonization with Candida species	1–2%	10–40%	Blood cultures Culture and histology of the biological sample	Echinocandins Azoles to be considered as a de-escalation therapy
*Aspergillus* spp.	Prolonged neutropenia, vascular amin use >24 h after surgery, ICU re-admission >1 bacterial infection, and high corticosteroid dosage	1–4%	60–90%	Possible/probable or certain diagnosis according to a combination of clinical, serological, radiological, histopathological, and microbiological factors	Voriconazole/ isavuconazole/liposomal amphotericin B
*Pneumocystis jirovecii*	Low CD4+ T-cell, CD8+ T-cell, and NK cell counts	2.1%	5–15%	Combination of radiological and clinical features, beta-d-glucan, PCR/immunofluorescence on bronchoalveolar lavage	Severe pneumonia: trimethoprim/sulfamethoxazole + Prednisone Pentamidine Primaquine + Clindamycin Mild disease: trimethoprim/sulfamethoxazole Dapsone Primaquine Atovaquone
*Cryptococcus* spp.	Use of calcineurin inhibitors and high-dose corticosteroids	0.32%		Cryptococcus antigen and culture of the sample	Induction: liposomal amphotericin B + Flucytosine Consolidation and Maintenance: fluconazole
Endemic fungi	Spelunking, farming, cleaning up bird droppings, and refurbishing buildings that have been inhabited by birds or bats, such as barns	≤3% in endemic areas	10–62%	Serologic tests, fungal culture, and PCR	Fluconazole or itraconazole for mild cases and liposomal amphotericin B for severe disease

## Data Availability

No new data were created or analyzed in this study.
